# Modeling the Impact of Excipients Selection on Nitrosamine Formation towards Risk Mitigation

**DOI:** 10.3390/pharmaceutics15082015

**Published:** 2023-07-25

**Authors:** Alberto Berardi, Maarten Jaspers, Bastiaan H. J. Dickhoff

**Affiliations:** DFE Pharma, Klever Str. 187, 47574 Goch, Germany; maarten.jaspers@dfepharma.com (M.J.); bastiaan.dickhoff@dfepharma.com (B.H.J.D.)

**Keywords:** MCC, nitrosamines, lactose, superdisintegrants, excipients

## Abstract

Risk control for nitrosamine impurities in drug products is currently a major challenge in the industry. Nitrosamines can form during drug product manufacturing and storage through the reaction of nitrites with amine-containing APIs or impurities. The level of nitrites in excipients and the rate of reaction often control the build-up of nitrosamine. Although the variability in nitrite levels across excipient types and suppliers is well recognized, the impact of excipient selection on the level of nitrosamine formed has not been systematically studied. This gap of knowledge is addressed in the current work. We present theoretical case studies of formulations where microcrystalline cellulose (MCC), or lactose supplier, or superdisintegrant type are changed in pursuit of lower levels of nitrite. The impact of the average, maximum, and minimum levels of nitrites in each excipient on nitrosamine formation in the dosage form is calculated. The input data for this calculation are the formulation composition, nitrosamine molecular weight (MW), percentage of conversion, and nitrite levels per excipient. The percentage of conversion (based on the formulation and manufacturing variables) and nitrite levels were taken from the recent literature. We show that changing the supplier of a single excipient, or of the three most critical excipients, can reduce nitrosamine formation by up to −59% and −89%, respectively. We also show that high-risk formulations, e.g., high MW nitrosamines, high dosage weights, and high percentages of conversion (e.g., wet granulation), can often be de-risked below regulatory acceptable daily intake via careful excipient selection. Finally, we provide an open-access tool that enables users to calculate the theoretical formation of nitrosamines in their specific formulations. This calculation template can be used for (i) the preliminary screening of the risk of nitrosamine formation in drug products and (ii) the preliminary assessment of the impact of excipient selection for risk mitigation.

## 1. Introduction

Nitrosamines are recognized to be probable or possible human cancerogenic substances and have been found in many pharmaceutical products [1,2]. Regulatory agencies worldwide have recently prompted marketing authorization holders (MAHs) to take action in relation to this health concern [3]. Both the Food and Drug Administration (FDA) and the European Medicines Agency (EMA) have issued guidance indicating the steps that MAHs should follow to evaluate the risk of nitrosamines, test for their presence, and eventually implement changes in pursuit of the reduction in nitrosamines in drug products. About 15% to 40% of all active pharmaceutical ingredients (APIs) are at risk of nitrosamine formation, and this challenge is shared across different compounds and pharmacological classes [4].

Nitrosamines are not directly cancerogenic, but require metabolic activation by certain enzymes of the P450 cytochrome family. Nitrosamines can be converted into alkylating agents (typically alkyldiazonium ions) that can interact with DNA [5]. Both metabolism and alkylation steps are covalent reactions that are typically influenced by steric and electronic interactions. Hence, the specific chemical features of a given nitrosamine can either decrease or increase their potency. A second aspect that could influence the cancerogenic potential of a nitrosamine is related to biological and pharmacokinetic complexities. Factors like absorption, distribution, clearance, and metabolism within physiological conditions could all influence the potency of a given nitrosamine. For example, aromatic N-nitrosamine can be subjected to Fischer–Hepp rearrangement under acidic conditions to form para-nitrosoarylamines. For a deeper understanding of the potency/mutagenicity of various nitrosamines, readers are referred to articles in the recent literature [4,6,7,8,9]. The topic of nitrosamine potency goes beyond the focus of this work. Here, we show the impact of excipient selection on nitrosamines formed during processing and tablet storage, with the objective of mitigating the risk to exceed acceptable daily intakes (ADIs) proposed by regulatory agencies.

Understanding the pathway to nitrosamine formation is key to developing appropriate mitigation strategies for nitrosamine reduction. Nitrosamines typically form through the reaction of secondary amines with nitrosating agents. Extensive research over the past few years has shown that secondary amines inherently present in APIs, present as impurities, or formed as degradation products of APIs can interact with nitrosating agents present in the formulation [10,11]. Nitrites that are present as impurities in excipients are the main source of nitrosating agents. The rate-limiting reagent in the formation of nitrosamines is typically the nitrite present at trace levels rather than the much more abundant secondary amine.

The formation of nitrosamines from nitrites in a drug product is often determined by two factors:

1. The level of nitrites in the formulation;
2. The percentage of conversion of nitrites into nitrosamines.

Risk assessments of drug products are usually conducted considering the worst-case scenario (i.e., the theoretical maximum levels) of both the nitrite levels and the extent of conversion (i.e., 100%) [12]. The risk is finally determined by comparing the calculated levels against the ADI for the nitrosamine of interest [3]. As the understanding of this topic is rapidly evolving, it is becoming apparent that the calculated nitrosamine content based on both the worst-case scenarios of nitrite levels in excipients and 100% conversion likely results in a largely overestimated risk compared to realistic conditions.

Moser et al. recently determined that the 100% conversion of nitrite into nitrosamines in the solid state is far from accurate [13]. In all cases studied, the level of N-nitrosamine formation in solid dosage forms plateaued at a level much lower than 100%. A maximum conversion of approximately 38% was measured in tablet dosage forms with the most favorable conditions for nitrosamine formation during stability tests. In all cases, one could argue that this worst-case scenario of 38% conversion of nitrites into nitrosamines is largely approximative. This is because the nitrite content in the formulations was based on the average levels reported in the literature, whereas the formation of nitrosamines was measured experimentally. Given the extremely high supplier-to-supplier variability of nitrites in excipients [12], the estimated nitrite level used in the article by Moser et al. might be vastly different from the actual nitrite content. Hence, the calculation of the conversion of nitrites into nitrosamines could, in turn, be approximative. Interestingly, Moser et al. also tested tablets spiked with larger and known quantities of nitrites and then measured the nitrosamine formation. The resulting calculation of the percentage of conversion was more realistic in this case, given that both the nitrite and nitrosamine contents were known experimentally. The highest percentage of conversion measured in the spiked formulations was 29%. It was also proven that the most favorable conditions for maximum conversion were (1) large excesses of secondary amine API; (2) API in the salt form; (3) wet granulation; and (4) storage in high-humidity conditions [13].

Moser et al. established, for the first time, a point of reference for the expected percentage of the conversion of nitrites into nitrosamines in tablets. In addition to the percentage of conversion, the second important factor determining nitrosamine content in a tablet is the nitrite content of excipients used. In a pioneering work, Schlingerman et al. recently showed a reduction in nitrosamine formation in a drug product via the selection of the excipient supplier providing the lowest level of nitrites [14]. However, so far, the impact of the selection of excipients with different levels of nitrite on the formation of nitrosamines has not been systematically studied [10,14]. As regulatory bodies indicate supplier qualification (e.g., a change of excipient supplier) and formulation design (e.g., a change of excipient type) as the main mitigation strategies to reduce nitrosamines [15], it is key to understand the extent that these strategies can reduce the risk of nitrosamine formation. Such an evaluation needs to take into account the realistic values of both nitrite levels in excipients and the percentage of conversion into nitrosamines (not 100%).

In this study, we calculate the theoretical nitrosamine content for model formulations using realistic percentages of conversion and the average content of nitrites, as indicated in the literature. Then, we calculate to what extent the nitrosamine content can change by replacing either the supplier of an excipient or the type of excipient in the formulation. The impact of specific variables such as the tablet dose weight, nitrosamine MW, and process of manufacturing (dry process vs. wet granulation) on nitrosamine formation and the consequent risk of exceeding regulatory thresholds is also evaluated. Finally, a calculation tool is provided whereby the user can directly calculate the contribution of each excipient on the theoretical formation of nitrosamine in a formulation. The input parameters for the calculation are the tablet weight and composition and nitrosamine MW. The percentages of conversion into nitrosamines, as well as the levels of nitrites in excipients, are provided based on the scientific literature [12,13].

The simulation presented here showcases to what extent the change of a single ingredient in a formulation can impact nitrosamines levels. Moreover, this article provides a preliminary framework for the preparation of nitrosamine risk assessments for drug products, which reflects the realistic values of both the nitrite content and the percentage of conversion.

## 2. Experimental Design

### 2.1. Case Studies on the Impact of Excipient Type and Supplier on Nitrosamine Formation

A series of case studies was designed to calculate the theoretical nitrosamine formation in a tablet based on the known values of:

- The level of nitrite in each excipient;
- The MW of the nitrosamine that is expected to form;
- The tablet dose weight;
- The expected percentage of conversion of nitrite into nitrosamine;
- The dosage form composition.

In each case study, the potential formation of nitrosamine (in ppm) per excipient was calculated from the nitrite levels and rate of conversion. Then, the contribution of each excipient on the final nitrosamine content in the tablet was calculated based on the tablet composition. The impact of the average, maximal and minimal levels of nitrites for selected excipients on the total nitrosamine formation was compared. The study design is detailed below.

#### 2.1.1. Theoretical Nitrosamine Formation per Excipient

The theoretical nitrosamine formation related to each excipient present in a dosage form was calculated according to Equation (1):

(1) $$Nitrosamine\;per\;excipient\;\left(\mathrm{ppm}\right)=\frac{Nitrite\;level\;in\;excipient\;\left(\mathrm{ppm}\right)\times MW\;of\;nitrosamine\;\left(\frac{g}{\mathrm{mol}}\right)\times \%\;of\;conversion}{MW\;nitrite}$$

This equation assumes that only a portion of the nitrites in the excipient will convert into nitrosamine in the presence of an amine upon tablet storage. The nitrite levels for each excipient were taken from the publication by Botzel et al. [12]. The values considered were the min., max. and mean, as representative of the best-, worst-, and average-case scenarios, respectively. The MW of the nitrosamine and the percentage of conversion used are described in Table 1. In the case of nitrosamine drug substance-related impurities (NDSRIs), an MW of 400 was used, as approximately representative of a median value [4]. In one case study, the formation of the small (low MW) nitrosamine N-Nitrosodiethylamine (NDEA), MW 102 g/mol, was assessed. The expected percentages of conversion of nitrite into nitrosamine were those reported by Moser et al. [13]. For NDSRIs, the percentages of conversion determined for the nitrite-spiked formulations were used, as those were accurately calculated based on the known concentrations of nitrites (as explained in the introduction). The percentages of conversion were selected in each case study based on the hypothetical amine type, form, and manufacturing process (direct compression (DC) or wet granulation (WG)). It must be noted that the percentages of conversion reported by Moser et al. were obtained under accelerated storage conditions, and in all cases, those percentages of conversion plateaued within the timeframe of the storage.

#### 2.1.2. Total Theoretical Formation of Nitrosamine in the Tablet

In each case study, the nitrosamine formation per excipient (ng) in the tablet was calculated according to Equation (2), based on the values of nitrosamines per excipient in ppm (Equation (1)) and the tablet weight and composition.

(2) $$Relative\;nitrosamine\;per\;excipient\;\left(\mathrm{ng}\right)=Tab\;wt\;\left(\mathrm{mg}\right)\times \%\;excipient\;\left(\frac{w}{w}\right)\times Nitrosamine\;per\;excipient\;\left(\mathrm{ppm}\right)$$

The tablet dose weights are indicated in Table 1. The compositions of the formulations are indicated below, in Table 2.

The chosen formulation compositions are realistic, and specifically, the DC formulation is similar to a previous one used in the literature [12]. The WG formulation contained a higher percentage of lactose than MCC, as is typical of many WG formulations [16]. The total nitrosamine formation and intake (ng) per tablet were finally calculated as the sum of the nitrosamine content for each of the excipients in the tablet. The contribution of the API to the nitrite content of the formulation was not considered.

The Excel template used for the calculations described in Equations (1) and (2) is presented as a Supplementary Materials file, together with an example formulation sheet. This template can be downloaded and used for theoretical nitrosamine risk evaluations of specific products.

### 2.2. Evaluation of Impact of Nitrosamine MW, Tablet Dose Weight, and Percentage of Conversion on Nitrosamine Formation

The impact of the nitrosamine MW, tablet dose weight, and percentage of conversion on the formation of nitrosamines was evaluated by comparing the average (average-case scenario), maximal (worst-case scenario), and minimal (best-case scenario) nitrite levels in MCC and lactose.

The absolute amount of nitrosamine formed increased linearly with increasing nitrosamine MW (based on Equation (1)), tablet dose weight (based on Equation (2)), and percentage of conversion (based on Equation (1)). The linear relation between each of these three factors (x-axis) and the theoretical nitrosamine formation (y-axis) are presented. One factor was plotted each time, keeping all other parameters fixed, as shown in Table 3. In each graph, three lines with different slopes are shown, corresponding to the formulations with the average, maximal, and minimal levels of nitrite in both MCC and lactose.

## 3. Results

### 3.1. Impact of Excipient Supplier Selection on the Formation of Nitrosamine Drug Substance-Related Impurities (NDSRIs)

Nitrosamine drug substance-related impurities (NDSRIs) are complex, API-related nitrosamines that are generated by nitrosation at the level of the whole drug substance. These nitrosamines are larger than the nitrosamines typically formed from amines present as impurities or degradation products (e.g., NDEA) [4]. Moser et al. recently indicated that amines present in tablets in low concentrations (e.g., 0.1% *w*/*w*), such as those present as impurity or degradation products, yielded a very low percentage of conversion into N-nitrosamines, with values often near or below the limit of quantification (LOQ) of the analytical method. This is unlike API-related amines, which are present in higher concentration and have shown a higher extent of conversion into NDSRIs [13]. The initial focus of this paper is therefore on NDSRIs, which seem to represent a higher risk due to the significantly higher percentage of conversion.

For most NDSRIs, no carcinogenic data are available. In the absence of these data, some regulatory authorities indicate an acceptable intake (AI) limit of 18 ng/day. However, the EMA has recently fixed an interim AI of 178 ng/day until a molecule-specific AI is defined [4,17]. Schlingemann et al. performed an in silico analysis of more than 12,000 small-molecule drugs and found that NDSRIs have molecular weights ranging from 200 to 1000 g/mol, with a median of approximately 400 g/mol. Based on this information, in our work, a hypothetical average case of NDSRIs with an MW of 400 was considered, and two AI limits were evaluated, i.e., 18 and 178 ng/day [4].

**Figure 1:**
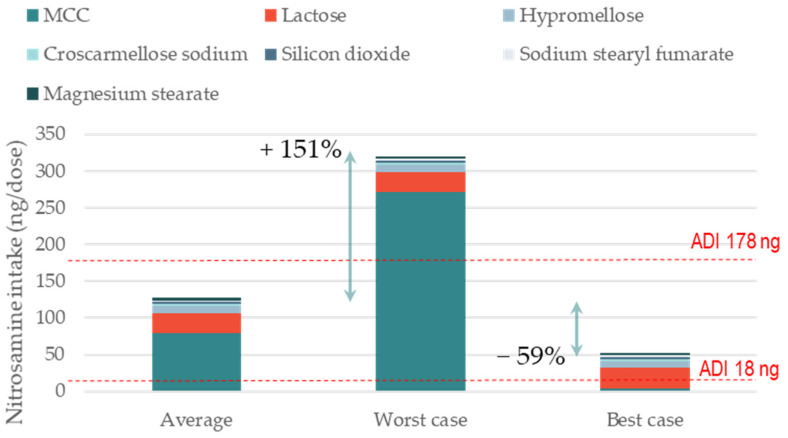
Impact of nitrite levels of MCC on the formation of a model nitrosamine. The three scenarios are representative of nitrosamine formation for a formulation where MCC contains average, highest, and lowest levels of nitrite. For all excipients other than MCC, average levels of nitrites were used and kept fixed; nitrosamine MW 400 g/mol; amine type secondary; amine form salt; tab weight 200 mg (single dose); manufacturing DC.

The first case study was a 200 mg directly compressed (DC) immediate-release tablet formulation containing 15% API and MCC, lactose, hypromellose, croscarmellose sodium, silicon dioxide, sodium stearyl fumarate, and magnesium stearate as excipients. Figure 1 shows the impact of changing a single excipient (i.e., MCC) supplier on the theoretical formation of nitrosamine. The average level of nitrites in each excipient, as well as the best- and worst-case scenarios of nitrites in MCC, were taken from the publication by Boetzel et al. [12]. A secondary amine API resulting in the formation of a nitrosamine with an MW of 400 was considered. The results show that the formulation with excipients containing an average level of nitrites was below the 178 ng/day AI limit of nitrosamine, but significantly exceeded the 18 ng/day. When MCC with the highest recorded level of nitrites was used, the nitrosamine levels in the tablet increased by +151%, well above the 178 ng/day AI limit. When MCC with the lowest level of nitrites was used, the theoretical nitrosamine formation was −59% lower (i.e., less than half) compared to MCC with average nitrite levels. The impact of changing to a supplier of MCC that has a low level of nitrites on de-risking a formulation towards nitrosamines is apparent.

A limitation of the approach described here is that the percentage of conversion was based on the value established by Moser et al. for a directly compressed formulation with the secondary amine API in the salt form [13]. The percentage of conversion used is, therefore, a realistic best estimate. Nevertheless, one should consider that even if the percentage of conversion is different from the one used, the relative increase and decrease in nitrosamine formation (i.e., +151% and −59%) for the worst- and best-case scenarios of MCC, compared to the average, still hold.

**Figure 2:**
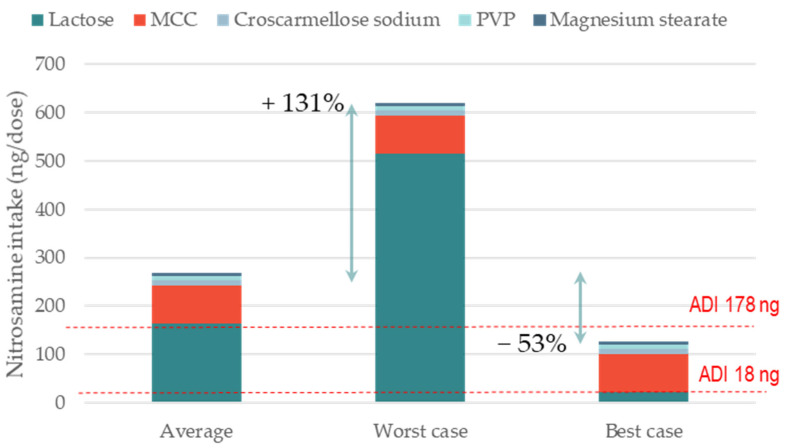
Impact of nitrite levels of lactose on the formation of a model nitrosamine. The three scenarios are representative of nitrosamine formation for a formulation where lactose contains average, highest, and lowest levels of nitrite. For all excipients other than lactose, average levels of nitrites were used; nitrosamine MW 400 g/mol; amine type secondary; amine form salt; tab weight 200 mg (single dose); manufacturing WG.

The second case study was a 200 mg wet granulated (WG) immediate-release tablet formulation containing 10% of a secondary amine API in the salt form. Lactose was the main component here, as is typical for WG formulations. In this case, the conversion of nitrites into nitrosamines was 29% (Table 1). This is higher than the percentage of conversion used for the DC formulation (i.e., 13%), as wet granulation promotes the distribution and mobility of both nitrites and amine salts, and therefore, increases the chance of contact and reactivity [13]. Figure 2 shows that both the average formulation and the worst-case formulation where lactose contains the highest nitrite content have levels well above the 178 ng/day ADI limit. The replacement of lactose in pursuit of a supplier with minimal nitrite levels would, in this case, reduces nitrosamine formation by −53% compared to the average formulation, which would be safely below the 178 ng/day ADI limit.

### 3.2. Impact of Re-Formulation on NDSRI Formation

**Figure 3:**
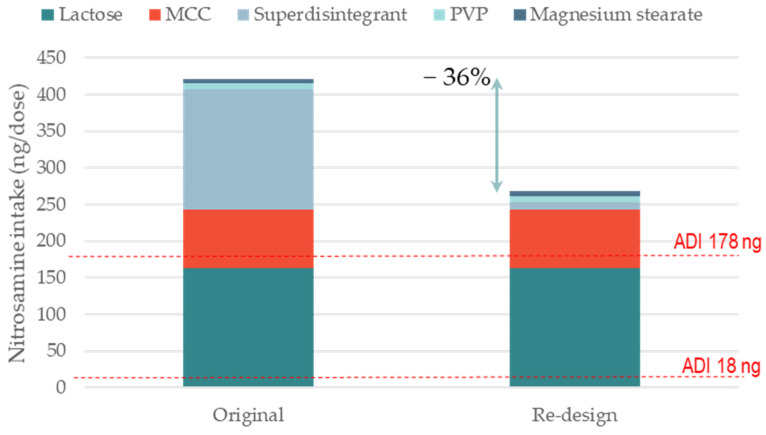
Impact of re-designing a formulation with a different type of superdisintegrant on the formation of a model nitrosamine. The two scenarios are representative of nitrosamine formation for two formulations: containing crospovidone (original) and croscarmellose sodium (re-design). Average levels of nitrites were used and kept fixed for all excipients; nitrosamine MW 400 g/mol; amine type secondary; amine form salt; tab weight 200 mg (single dose); manufacturing WG.

**Figure 4:**
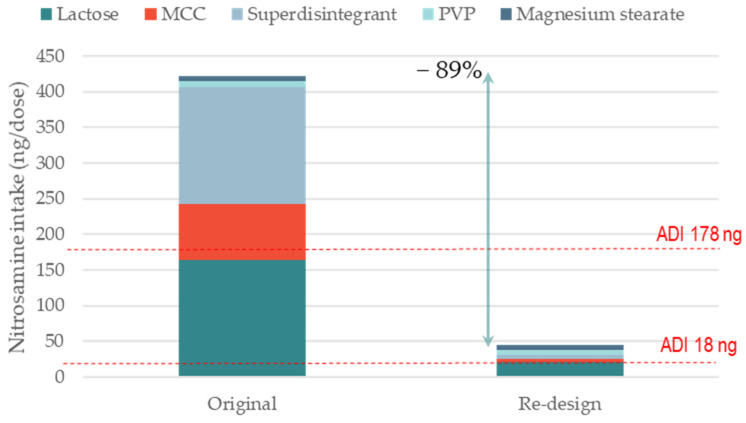
Impact of cumulative effect of (i) selecting MCC and lactose supplier and (ii) re-designing a formulation with a different type of superdisintegrant on the formation of a model nitrosamine. The two scenarios are representative of nitrosamine formation for two formulations with average level of nitrites for crospovidone, MCC, and lactose (original) and lowest levels of nitrites for croscarmellose sodium, MCC and lactose (re-design); nitrosamine MW 400 g/mol; amine type secondary; amine form salt; tab weight 200 mg (single dose); manufacturing WG.

Both aforementioned cases are examples of nitrosamine risk mitigation via the qualification of a different excipient supplier for the major filler excipient. An alternative risk mitigation strategy is the re-formulation of a product using excipient types that contain lower levels of nitrites. Figure 3 shows the impact of changing the superdisintegrant on nitrosamine formation. The model formulation (Table 2) is similar to that shown in Figure 2 (i.e., WG with secondary amine in the salt form). The original product contained crospovidone, which had average nitrite levels of 6.5 ppm. The re-formulated product contained croscarmellose sodium, with average nitrite levels of 0.42 ppm. The nitrosamine formation could be reduced by −36% using this reformulation strategy. Although the superdisintegrant made up only 5% of the tablet content, its replacement could lead to a significant reduction in the theoretical nitrosamine content. This is because the nitrites per unit weight level is much higher for crospovidone than for other disintegrants [12]. In this specific case study, however, the superdisintegrant replacement with croscarmellose sodium was not sufficient to completely de-risk the formulation towards nitrosamine levels below the 178 ng/day ADI limit. For this reason, in the next exercise (Figure 4), we evaluated the cumulative impact of (i) selecting MCC and the lactose supplier based on the lowest nitrite levels and (ii) replacing crospovidone with croscarmellose sodium on the formation of a model nitrosamine. In this case, the complete reformulation strategy enabled a reduction in nitrosamine formation of −89% (from 422 to 45 ng per dose). The fully reformulated product, therefore, appeared to be de-risked with respect to the 178 ng/day ADI limit.

### 3.3. Impact of Nitrosamine MW and Tablet Dose Weight on Nitrosamine Formation

**Figure 5:**
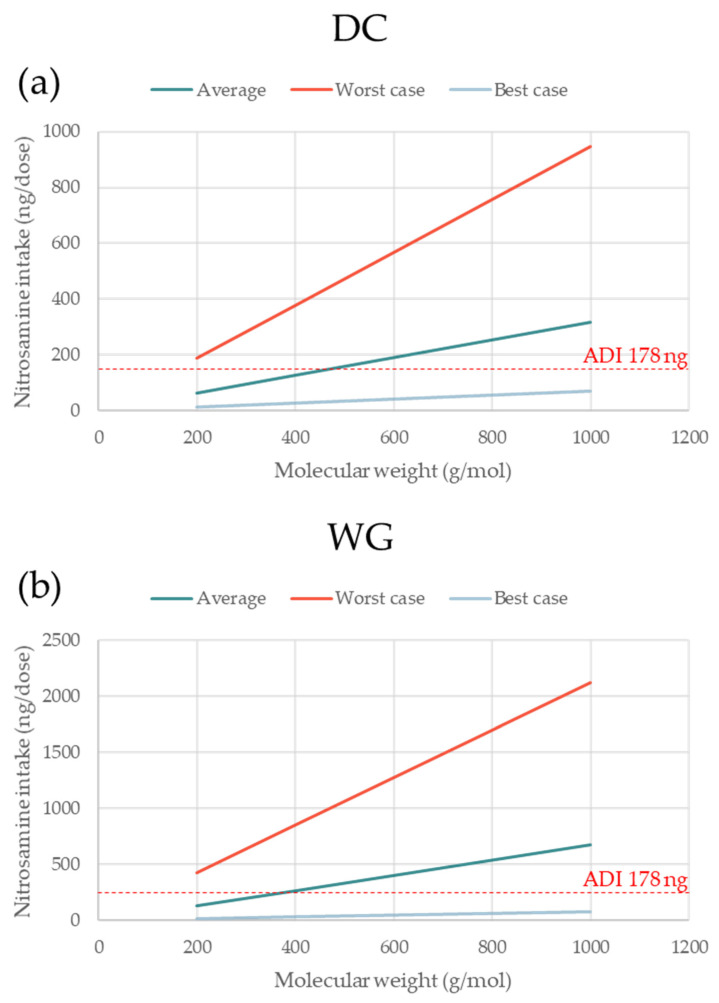
The effect of nitrosamine MW on nitrosamine intake for formulations based on excipients with average, maximal, and minimal nitrite levels. (**a**,**b**) represent the direct compression and wet granulated formulations, respectively. The three scenarios are representative of nitrosamine formation for formulations where both MCC and lactose contain average, highest, and lowest levels of nitrite; amine type secondary; amine form salt; tab weight 200 mg (single dose).

NDSRIs with higher MW could be expected to have lower carcinogenic potential. However, until carcinogenic data for a specific nitrosamine are available, the 178 ng/day and 18 ng/day limits apply regardless of the MW [4]. It is, therefore, important to visualize to what extent the MW of the nitrosamine can impact the theoretical amount of nitrosamine formed. Figure 5a,b shows the levels of nitrosamine build-up for the model DC and WG formulations containing the average, highest, and lowest levels of nitrites for both fillers, MCC and lactose. As expected, based on the different percentages of conversion, the WG formulation has typically higher levels of nitrosamine than the DC one. When using excipients with minimal nitrite levels, the amount of nitrosamine remains safely below the 178 ng/day threshold across the full span of MW for both formulations. On the other hand, the worst-case formulations would yield nitrosamine values well above the 178 ng/day limit at any MW. For the formulations with average nitrite content, meeting the 178 ng/day requirement is dependent on the MW of the nitrosamine formed. The fact that the slopes of the three curves are directly proportional to the levels of nitrite in the formulations indicates that formulations low in nitrites are less sensitive to the API MW (and consequently, to the nitrosamine MW). It is evident that the high molecular weight of the API (and nitrosamine) is a much greater risk factor for formulations that have a high nitrite content.

**Figure 6:**
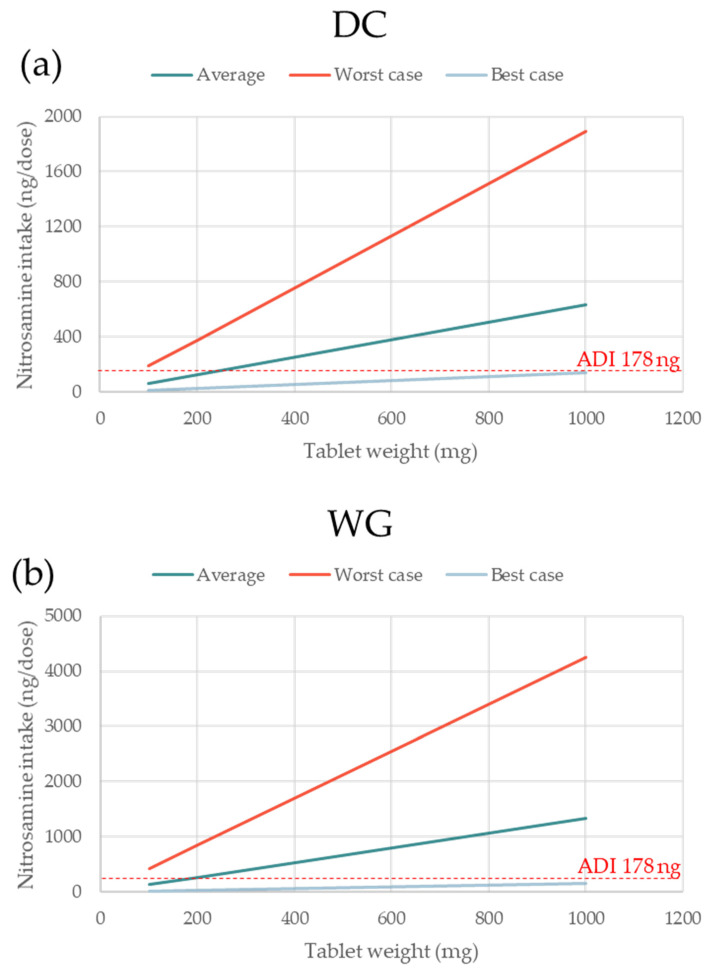
The effect of tablet weight on the formation of a nitrosamine for formulations based on excipients with average, maximal, and minimal nitrite levels. (**a**,**b**) represent the direct compression and wet granulated formulations, respectively. The three scenarios are representative of nitrosamine formation for formulations where both MCC and lactose contain average, highest, and lowest levels of nitrite. For all excipients other than MCC and lactose, average levels of nitrites were used and kept fixed; nitrosamine MW 400 g/mol; amine type secondary; amine form salt.

It is intuitive that the weight of the oral solid dosage form also has a linear correlation with the theoretical formation of nitrosamines. Even in this case (Figure 6), formulations containing low levels of nitrites guarantee a better control of the nitrosamine formation across all dose weights. For a dosage form weight of up to 1000 mg, both the DC and WG formulations containing MCC and lactose with the lowest levels of nitrites would lead to nitrosamine levels below 178 ng/dose.

### 3.4. Impact of the Percentage of Conversion on NDSRIs Formation

Moser et al. showed that, in the solid state, a 100% conversion of nitrites into nitrosamines is extremely unlikely. This is because in the solid state (unlike liquids), the diffusion and mobility of compounds is limited. Hence, it is expected that not all nitrites present in the dosage form will come into contact with amines [13]. The highest percentage of conversion measured in that study was 38%.

**Figure 7:**
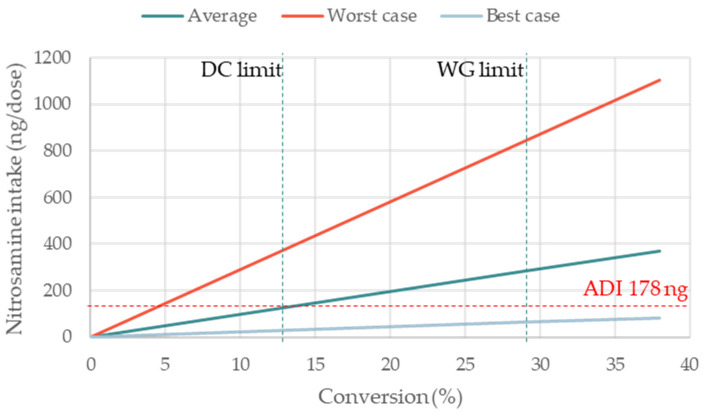
Impact of nitrite levels of MCC and lactose on the formation of a nitrosamine at increasing percentages of conversion. The three scenarios are representative of nitrosamine formation for formulations where both MCC and lactose contain average, highest, and lowest levels of nitrite; nitrosamine MW 400 g/mol; tab weight 200 mg (single dose). Vertical dash lines indicate upper limit of percentage of conversion for spiked DC and WG formulations, as indicated by [13].

Throughout this investigation, we used fixed percentages of conversion based on the most realistic information available to date. Nevertheless, one would expect that the percentages of conversion will fluctuate case by case, based on the specific formulations and conditions of manufacturing and storage. For example, it has been reported that even the salt form of nitrite (e.g., sodium or ammonium) can have a high impact on the percentage of conversion into nitrosamine [18]. To gain a broader understanding, we plotted the impact of the percentage of conversion on the formation of nitrosamines, always comparing formulations where MCC and lactose had the highest, average, and lowest levels of nitrites (Figure 7). It is apparent that the selection of MCC and lactose suppliers with the lowest levels of nitrites provided much better chances to meet the regulatory limit of nitrosamine intake. Once again, the nearly horizontal slope for the low nitrite formulation (e.g., best case) indicates that the formation of nitrosamines would remain nearly insensitive to high percentages of conversion.

Overall, the best-case scenario, i.e., filler excipients with the lowest levels of nitrites in the market (nitrite ≈ 0.04–0.07 ppm), appeared to effectively de-risk formulations towards the 178 ng/day ADI of nitrosamines, even in the highest-risk cases (i.e., high MW nitrosamine, high tablet dose weight, and high percentage of conversion).

### 3.5. Impact of Excipient Supplier Selection on the Formation of Low-Molecular-Weight Nitrosamines

**Figure 8:**
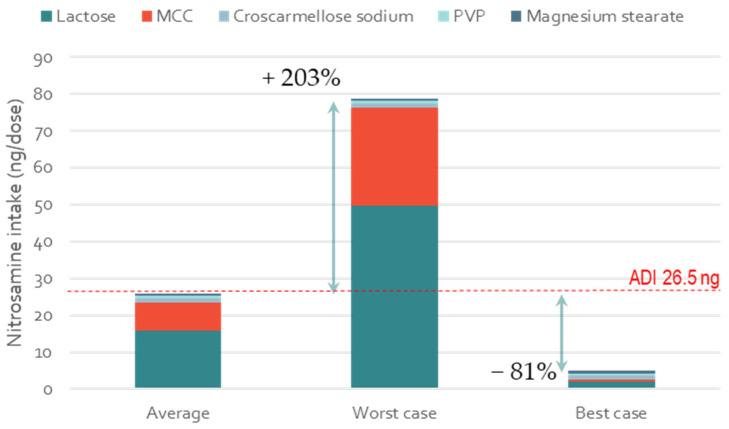
Impact of excipient selection (MCC and lactose) on the formation of NDEA. The three scenarios are representative of nitrosamine formation for a formulation where MCC and lactose contain average, highest, and lowest levels of nitrite. For all excipients other than lactose, average levels of nitrites were used and kept fixed; nitrosamine MW 102 g/mol; tab weight 1000 mg (single dose); manufacturing WG.

In addition to NDSRIs, small and potent nitrosamines that are typically formed from low MW amine impurities are also a major concern. Figure 8 shows the theoretical levels of N-Nitrosodiethylamine (NDEA) formed in the model WG formulation using MCC and lactose with the average, highest and lowest levels of nitrites. Given the high potency of this nitrosamine and the tight regulatory limit of both the FDA and EMA of 26.5 ng/day ADI [11,17], the low nitrite formulation would be preferred to de-risk this product. It is worth noting that the percentage of conversion used here was only 2.2%, as this was the percentage of conversion found by Moser et al. for amine compounds present in low concentrations (e.g., 0.1%, as amine impurities would be). In this case, the percentage of 2.2% is potentially approximative because it is based on assumed levels of nitrite and not on known spiked concentrations. Carloni et al. recently estimated similar percentages of the conversion of nitrite into nitrosamine for amine (dimethylamine substrate) incubated in a physical blend with MCC [18]. If the percentage of conversion was higher than 2.2% in other realistic formulations, the risk of NDEA formation would be superior, and the excipient supplier selection would play an even more important role. Nevertheless, the relative increase and decrease in nitrosamine intake for the worst- and best-case formulations (i.e., +203 and −81%) would remain the same, regardless of the percentage of conversion.

### 3.6. A Tool for the Calculation of Nitrosamine Formation in Oral Solid Dosage

Risk evaluation of the nitrosamine formation in a drug product remains a major challenge. The kinetics and factors impacting the formation of nitrosamines in oral solid dosage forms are becoming increasingly better understood [4,13]. On these bases, we developed a simple Excel tool for the calculation of nitrosamines’ formation in oral solid dosage (Supplementary Materials File—toolkit). The input data that must be inserted for the analysis are:

- The tablet dose weight;
- The dosage form composition (in percentage);
- The level of nitrite in each ingredient;
- The MW of the nitrosamine that is expected to form;
- The expected percentage of conversion of nitrite into nitrosamines.

The estimated values of nitrite levels in the selected excipients and the percentages of conversion can be found in two separate sheets of the Excel file. Specifically, the average, highest, and lowest levels of nitrites for some common excipients are reported based on the publication by Botzel et al. [12]. The percentages of conversions for DC and WG formulations for secondary amine APIs both in salt and base form are reported on a separate sheet, based on the publication of Moser et al. [13]. It must be noted that the percentages reported are those related to NDSRIs.

To evaluate the impact of a change of excipient (type or supplier) on the nitrosamine formation, two options of formulations are given, namely “original” and “excipient selection”. The output of the calculation is the quantity of nitrosamine formed in each formulation, broken down per the contribution of each excipient (percentage). For an even more accurate assessment, rather than using the literature data, the levels of nitrites in each excipient and percentages of conversion can be determined experimentally for the specific dosage form and subsequently inserted into the Excel file.

## 4. Conclusions

In this study, we showed the impact of excipient supplier selection and the reformulation of superdisintegrant on the theoretical formation of nitrosamine in drug products. A change of supplier and/or a reformulation of critical excipients proved to substantially reduce the theoretical formation of nitrosamines at fixed percentages of reaction. High-MW nitrosamine, a high tablet dose weight, and a high percentage of conversion (e.g., WG formulation of secondary amine APIs in the salt form) were at the highest risk of exceeding the ADIs set by the regulatory agencies. It was shown, however, that nitrosamine levels in low-nitrite formulations remained under control even for high-risk products. It appears evident that the supplier selection of filler excipients, such as MCC and lactose, and the replacement of critical excipients, such as crospovidone, are obvious nitrosamine risk-mitigation strategies. The same principle can be applied to other excipients beyond the case studies presented here.

Finally, to enable other scientists to estimate the formation of nitrosamine in their specific formulations, we present an open-access Excel template for simple calculation. Essential variables, such as nitrite levels and the percentages of conversion, are provided based on the most recent scientific literature. For higher accuracy in the calculations, users of the tool could replace the literature values with experimental ones. The Excel tool might be used by formulators and regulatory departments for the preliminary screening of the risk of nitrosamine formation in specific formulations. The tool also facilitates the evaluation of the impact of a change of excipient (type or supplier) on nitrosamine formation towards showcasing evidence-based risk mitigation.

It is worth pointing out that while the Excel template provides an easy and rapid tool to showcase risk mitigation, it is not a substitute for a more extensive risk analysis based on experimental data on nitrosamine, and where all risk factors are weighed out. For instance, one aspect that is overlooked in this model and that has not yet been thoroughly investigated by the scientific literature is the uneven distribution and availability of nitrite within different type of excipients. In the simplified model used here, all nitrites in the excipients were considered equally available to convert into nitrosamine.

This study shows that excipient types and suppliers with the lowest levels of nitrites currently available on the market (i.e., 0.04 to 0.1 ppm) could reduce nitrosamines in the model formulations by up to nearly 10-fold compared to formulations with average levels of nitrites. Such a drastic reduction in nitrosamines appears to efficiently de-risk most, if not all, formulations towards the 178 ng/day ADI limit for nitrosamines. A reduction in nitrosamines of up to 10-fold, such as that obtained by careful excipient selection, might not always be sufficient to lower nitrosamine below the 18 ng/day ADI limit. This is particularly the case for high-risk formulations (high-MW nitrosamine, high tablet dose weight, and high percentage of conversion). In such challenging cases, additional mitigation strategies (alongside excipient selection) would be recommended to lower nitrosamines below the 18 ng/day ADI limit. Such additional mitigation strategies could include (i) a reduction in the percentage of conversion of nitrites into nitrosamines by selecting a dry method of manufacturing, e.g., direct compression, and/or (ii) the addition of nitrite scavengers in the formulation [9,19].

It must be mentioned that, during the publication of this article, the EMA issued an update on the questions and answers document on nitrosamines (on the 7 July 2023) [20]. In this update, a new approach to categorize the carcinogenic potency for N-nitrosamines based on structural features was introduced. Based on the potency category of a given nitrosamine, a different ADI will apply. Depending on the category, ADI thresholds could be 1500, 400, 100, or 18 ng/day. The findings of the current article remain valid following this update. In fact, particularly for high-risk formulations, the risk of exceeding the 400, 100, and 18 ng/day limits remains high. Hence, the opportunity to reduce nitrosamine via the control of the excipient type and supplier remains highly relevant, as are all model calculations presented in the current manuscript.

The reduction in nitrosamines via the selection of excipients low in nitrite appears valuable. However, the reliability of this mitigation strategy is dependent on the possibility of sourcing excipients with consistently low levels of nitrites. Going forward, drug product manufacturers might request excipient vendors to supply excipients not only low in nitrites, but with a proven consistency of low nitrite levels. It is expected that the successful adaptation of current formulations and the development of new drug products low in nitrosamines will require close collaboration between the drug producers and excipient manufacturers. To tackle this challenge, the joint support of regulatory, quality, R&D formulation and R&D analytical departments from both the pharmaceutical and excipient industries is necessary.

## Figures and Tables

**Table 1:** Case study descriptions, definitions of variables, and percentages of conversion used (based on [13]).

Nitrosamine Studied	Case Study of Replacement	Nitrosamine MW	Tablet Dose Weight (mg)	Amine Type	Amine Form	Manufacturing	Expected Percentage of Conversion
Nitrosamine drug substance-related impurity (NDSRI)	MCC supplier	400	200	2nd	Salt	DC	13%
	Lactose supplier	400	200	2nd	Salt	WG	29%
	Superdisintegrant type	400	200	2nd	Salt	WG	29%
	MCC + lactose supplier + Superdisintegrant type and supplier	400	200	2nd	Salt	WG	29%
Small nitrosamine N-Nitrosodiethylamine (NDEA)	MCC + lactose supplier	102	1000	2nd	Salt	WG	2.2%

**Table 2:** Formulation compositions. Percentages are *w*/*w*.

	NDSRI Case Studies				NDEA Case Study
	MCC Supplier	Lactose Supplier	Superdisintegrant Type	MCC + Lactose Supplier + Superdisintegrant Type and Supplier	MCC + Lactose Supplier
**Manufacturing**	DC	WG	WG	WG	WG
**Ingredients**					
API	15%	10%	10%	10%	10%
MCC	50%	22.5%	22.5%	22.5%	22.5%
Lactose	22.5%	60%	60%	60%	60%
Croscarmellose sodium	3%	5%	5% (either/or)	5% (either/or)	5%
Crospovidone	-	-			-
Hypromellose	5%	-	-	-	-
Povidone	-	2%	2%	2%	2%
Silicon dioxide	1%	-	-	-	-
Sodium stearyl fumarate	3%	-	-	-	-
Magnesium stearate	0.5%	0.5%	0.5%	0.5%	0.5%

**Table 3:** Fixed and variable parameters used in the evaluation of the impact of nitrosamine MW, tablet dose weight, and percentage of conversion on nitrosamine formation.

Parameter	Impact of Nitrosamine MW	Impact of Dose Weight	Impact of Percentage of Conversion
Nitrosamine MW (g/mol)	Variable	400	400
Tablet dose weight (mg)	200	Variable	200
Amine type	2nd	2nd	Variable
Amine form	Salt	Salt	Variable
Manufacturing	DC/WG	DC/WG	Variable
Percentage of conversion	13%/29%	13%/29%	Variable

## Data Availability

Not applicable.

## Supplementary Materials

The following supporting information can be downloaded at: https://www.mdpi.com/article/10.3390/pharmaceutics15082015/s1, Table S1: Excel tool to estimate the theoretical nitrosamine formation in a drug product.
